# Prognostic Value of Homotypic Cell Internalization by Nonprofessional Phagocytic Cancer Cells

**DOI:** 10.1155/2015/359392

**Published:** 2015-10-04

**Authors:** Manuela Schwegler, Anna M. Wirsing, Hannah M. Schenker, Laura Ott, Johannes M. Ries, Maike Büttner-Herold, Rainer Fietkau, Florian Putz, Luitpold V. Distel

**Affiliations:** ^1^Department of Radiation Oncology, University Hospital Erlangen, 91054 Erlangen, Germany; ^2^Department of Pathology, University Hospital Erlangen, 91054 Erlangen, Germany

## Abstract

*Background*. In this study, we investigated the prognostic role of homotypic tumor cell cannibalism in different cancer types. *Methods*. The phenomenon of one cell being internalized into another, which we refer to as “cell-in-cell event,” was assessed in 416 cases from five head and neck cancer cohorts, as well as one anal and one rectal cancer cohort. The samples were processed into tissue microarrays and immunohistochemically stained for E-cadherin and cleaved caspase-3 to visualize cell membranes and apoptotic cell death. *Results*. Cell-in-cell events were found in all of the cohorts. The frequency ranged from 0.7 to 17.3 cell-in-cell events per mm^2^. Hardly any apoptotic cells were found within the cell-in-cell structures, although apoptotic cell rates were about 1.6 to two times as high as cell-in-cell rates of the same tissue sample. High numbers of cell-in-cell events showed adverse effects on patients' survival in the head and neck and in the rectal cancer cohorts. In multivariate analysis, high frequency was an adverse prognostic factor for overall survival in patients with head and neck cancer (*p* = 0.008). *Conclusion*. Cell-in-cell events were found to predict patient outcomes in various types of cancer better than apoptosis and proliferation and might therefore be used to guide treatment strategies.

## 1. Background

The mechanism by which a cell becomes internalized into another is the definition of the term “cell-in-cell formation” [1]. Cell-in-cell (CIC) formation includes the internalization of living lymphocytes into nonphagocytic cells (emperipolesis), the homotypic and heterotypic phagocytosis-like uptake of living or dead cells, and the invasion of one tumor cell into the host cell (entosis). The definition also covers the fate of the included cells, which can undergo cell death, remain in the host cell, undertake cell division, or pass out of the cell. In this context the term cell cannibalism arose that describes the uptake of alive and dead tumor cells as well as of lymphocytes by tumor cells and is restricted to cancer cells [2]. Cannibalism was suggested as a marker of malignancy [3] and reported to provide an advantage for survival. Lugini et al. reported that cannibalistic malignant metastatic melanoma cells engulf autologous live CD8^+^ T-lymphocytes as a source of nutrients under starvation conditions [4]. Reports on homotypic cannibalism are conflicting, proposing a tumor-promoting and metastasis-protecting role. This discrepancy complicates the use of homotypic cannibalism as a prognostic factor [5–7] and highlights that further studies are necessary to shed light on this phenomenon and its impact on eventual cancer type-specific prognosis. Investigation on the phagocytic activity of cancer cells and normal fibroblasts may contribute to providing new insights into this issue [8, 9]. The use of in vivo experiments to identify the triggering factors and underlying mechanisms of cell cannibalism may facilitate a prognosis-relevant examination of CIC formation in association with specific proteins in tumor tissue sections.

Here, we present data on the prognostic relevance of CIC structures and their divergent effects on patient prognosis in three different tumor entities. Additionally, the impact of CIC rates was compared to those of apoptosis and proliferation.

## 2. Methods

### 2.1. Human Specimens

Cancer tissue samples from 416 patients were evaluated for the presence of CIC structures. Patients originated from seven different cohorts. (i) One cohort of anal carcinomas (*n* = 23) and (ii) one rectal cancer cohort (*n* = 83) were studied. Five head and neck squamous cell carcinoma (HNSCC) cohorts were compared, including (iii) an early disease, low-risk group (*n* = 62); (iv) an advanced disease, high-risk group (*n* = 52); (v) cancer biopsies with affected lymph nodes (*n* = 29); (vi) pretherapeutic biopsies with posttherapeutic tumor resections (*n* = 35); and (vii) pretherapeutic tumor resections of the central tumor area and the invasion front (*n* = 143). All patients were treated by definitive, adjuvant, or neoadjuvant radiotherapy or radiochemotherapy. Patients characteristics are described in Tables 1 and 2. Patient characteristics of five of the cohorts were previously published [10–13]. All samples were processed into tissue microarrays (TMAs) of 1.6–2 mm diameter cores [14]. Clinical data were obtained from the Erlangen Tumour Centre Database. All patients signed a “front door” informed consent allowing collection of their tissue and clinical data. The study was approved by the Ethics Review Committee of the University Hospital Erlangen, Erlangen, Germany.

### 2.2. Antibodies and Immunohistochemistry

Immunohistochemistry was performed for detection of specific proteins in tissue sections using several antibodies including Ki-67 (DakoCytomation, Hamburg, Germany), cleaved caspase-3 (Cell Signaling, Danvers, MA, USA), E-cadherin (BD, Heidelberg, Germany), CD68 (DakoCytomation), and Alexa Fluor 488, 555, 594, and 647 conjugated secondary antibodies (all from Invitrogen, Darmstadt, Germany).

Double staining was performed with Ki-67 and cleaved caspase-3-specific antibodies. Briefly, sections were incubated overnight with a cleaved caspase-3-specific antibody and then a biotin-labeled secondary antibody. Biotin was visualized using streptavidin-biotinylated alkaline phosphatase complex (DakoCytomation). Fast Red was used as a chromogen. A double staining enhancer (Zymed, San Francisco, California, USA) was used followed by an avidin and biotin block (Avidin/Biotin Blocking Kit, Vector Laboratories, Peterborough, UK). Slides were incubated with Ki-67-specific primary antibodies after application of a postblock solution. Secondary antibodies covalently linked with an AP-Polymer (ZytoChem-Plus, Berlin, Germany) were used with Fast Blue (Sigma-Aldrich, Taufkirchen, Germany) as a chromogen. E-cadherin labeling was performed on a Ventana BenchMark Ultra stainer (Roche, Grenzach-Wyhlen, Germany) using CC1 buffer (Benchmark ULTRA CC1, Roche) for antigen retrieval. Cell nuclei were stained with hematoxylin.

In a quadruple immunofluorescence approach, four antigens were labeled successively in HNSCC tumor specimens. Antigen retrieval was performed in a steam cooker (Biocarta Europe, Hamburg, Germany) for 5 min at 120°C using a target retrieval solution (pH 6) (TRS6, DakoCytomation) or 0.01 M Na-citrate buffer (pH 6). Cells were stained with primary and secondary antibodies, nuclei were counterstained with 4′,6-diamidino-2-phenylindole (DAPI) (Roche), and slides were mounted with Vectashield medium (Vector Laboratories).

### 2.3. Imaging and Image Analysis

Stained TMAs were scanned with a high throughput scanner (Mirax MIDI Scan, Zeiss, Göttingen, Germany) equipped with a Plan-Apochromat objective (20x; NA: 0.8, Zeiss) and a camera (Stingray F146C, AVT, Stadtroda, Germany). Images were converted into TIF format and each TMA spot was saved separately. CIC structures were counted using Biomas image processing software (MSAB, Erlangen, Germany). A minimum of two TMA core sections with tumor areas of 0.75 mm^2^ was evaluated per patient. Apoptotic cells, proliferating cells, and CIC structures were counted in the same area. To achieve this, the E-cadherin TMA spot was precisely aligned with Ki-67/cleaved caspase-3 double-stained spot using the image analysis system. The region of interest was selected in the E-cadherin spot and transferred to the Ki-67/cleaved caspase-3 spot, and events were counted.

### 2.4. Statistical Analysis

IBM SPSS Statistics version 19 was used. The Kolmogorov-Smirnov test and Lilliefors test were applied for testing normality. The local failure-free, metastasis-free, and overall survival was calculated according to Kaplan Meier. The log-rank test was used to compare survival curves between subgroups of patients. Univariate and multivariate regression analyses of overall survival were performed using Cox's proportional hazards model (Table 3, additional file 2, Tables  A2-A3 in Supplementary Material available online at http://dx.doi.org/10.1155/2015/359392). The proportional hazards assumption was tested through plotting log-minus-log curves. *p* values < 0.05 were considered to be significant.

## 3. Results

### 3.1. Study Groups

The frequency and prognostic relevance of CIC structures in tumor tissue were investigated. A total of 416 tumor tissue samples from five cohorts of HNSCC and one (i) anal and one (ii) rectal cancer cohort were analyzed for the presence of CIC structures. The five HNSCC cohorts consisted of the following: (iii) a low-risk, early disease, treated by adjuvant radiochemotherapy (RCT); (iv) a high-risk, advanced disease, treated by definitive radiotherapy (RT) [10]; (v) metastatic disease, treated by adjuvant RT or RCT [11]; (vi) no distant metastasis, treated by neoadjuvant RCT [12]; and (vii) tumors treated by adjuvant RT or RCT having tissue samples of the central tumor area and the invasion front. The patients' clinical and histological characteristics are depicted in Tables 1 and 2. Overall survival in the head and neck and rectal and anal cancer cohort were 60.3%, 75.0%, and 68.5% at 5 years, respectively. The median follow-up in the head and neck and rectal and anal cancer cohort were 4.6, 7.2, and 3.8 years, respectively. Median and mean local failure-free, metastasis-free, and overall survival time are presented in additional file 2, Table A1.

### 3.2. Criteria of CIC Structures

**Figure 1:**
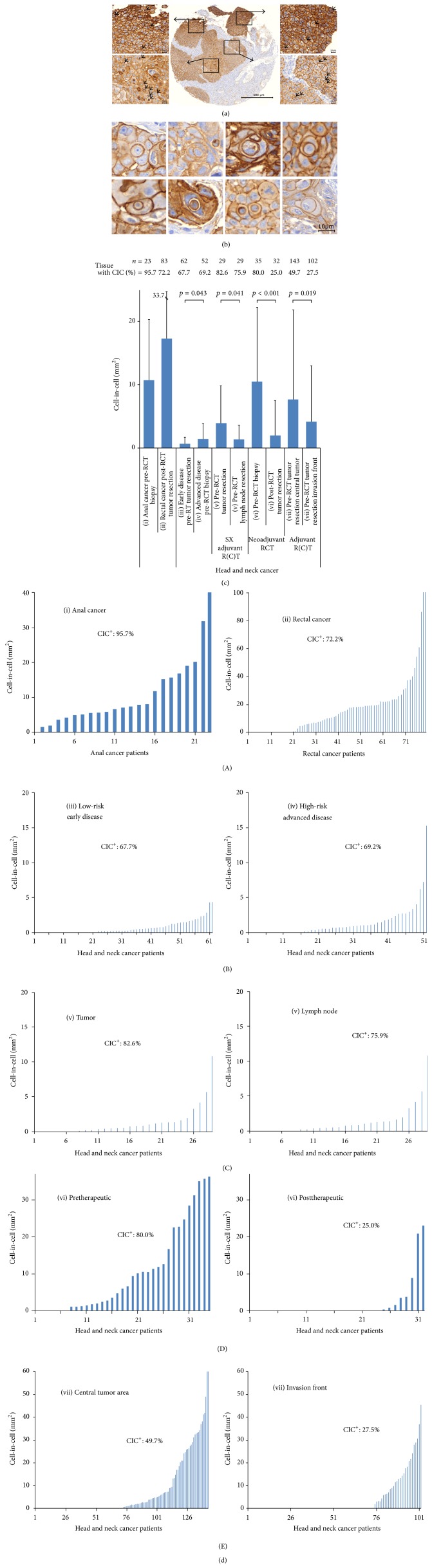
Cell-in-cell structures are found in different tumor tissues. (a) Representative image of an E-cadherin-labeled head and neck squamous cell carcinoma tissue microarray spot with numerous CIC structures and magnifications of indicated regions. (b) Representative images of E-cadherin-labeled head and neck cancer CIC structures. (c) Frequency of CIC structures in different cancer types. (d) Comparative frequency of CIC (A) in the tumor tissue of rectal and anal cancer patients, (B) in low-risk and high-risk HNSCC patients, (C) in the primary tumor of HNSCC tumors and the affected regional lymph nodes, (D) before and after neoadjuvant radiochemotherapy, and (E) in the central tumor area and invasive front of HNSCC. R(C)T: radiochemotherapy or radiotherapy.

All tumor tissues were processed into TMAs and were stained for the adhesion molecule E-cadherin to visualize cell membranes. Nuclei were counterstained with hematoxylin (Figure 1(a)). Stained TMA spots were analyzed for the presence of CIC events per mm^2^ using an image analysis system. Three criteria were used to define homotypic CICs: total encirclement of the inner cell by the host cell membrane, a semilunar host cell nucleus, and a round shape of the inner cell (Figure 1(b)).

### 3.3. CIC Structures Are Found in Various Tumor Tissues with Varying Frequencies

CIC structures were found in all of the cancer cohorts. The average frequency ranged from 0.7 CICs/mm^2^ in the (iii) low-risk, early disease HNSCC to 17.3 CICs/mm^2^ in the (ii) rectal cancer group (Figure 1(c)). The percentage of CIC-positive cancer tissues varied from 25.0% in the (vi) posttherapeutic HNSSC collection to 95.7% in the (i) anal cancer cohort (Figure 1(d) (A)–(E)). The pretherapeutic biopsies from the anal cancer cohort showed a high frequency of CIC-positive samples, with an average frequency of 10.7 CICs/mm^2^. However, the highest single values of up to 200 CICs/mm^2^ were observed in the (ii) posttherapeutic rectal cancer cohort.

In HNSCC tumor samples, we grouped the patients cohorts according to low- and high-risk characteristics and treatment modalities for separate analyses and comparison. We found that (iv) high-risk, inoperable patients with advanced disease had significantly more CIC than (iii) low-risk patients with early disease (Figures 1(c) and 1(d) (B)). In (v) lymph nodes, the average CIC numbers were significantly lower than in the corresponding primary tumors, while the number of CIC-positive patients differed only slightly (Figures 1(c) and 1(d) (C)). In a HNSCC cohort with (vi) pretherapeutic biopsy and tumor resections six weeks after RCT, both the frequency of CIC-positive patients and the average CIC number decreased significantly (Figures 1(c) and 1(d) (D)). We also compared CIC numbers in the (vii) central tumor area and at the invasive front. In the central tumor area, more samples were CIC-positive and the overall average value of CIC structures was higher than in the invasive front (Figures 1(c) and 1(d) (E)).

### 3.4. CIC Rate Compared to Apoptotic Cell Death and Proliferation

**Figure 2:**
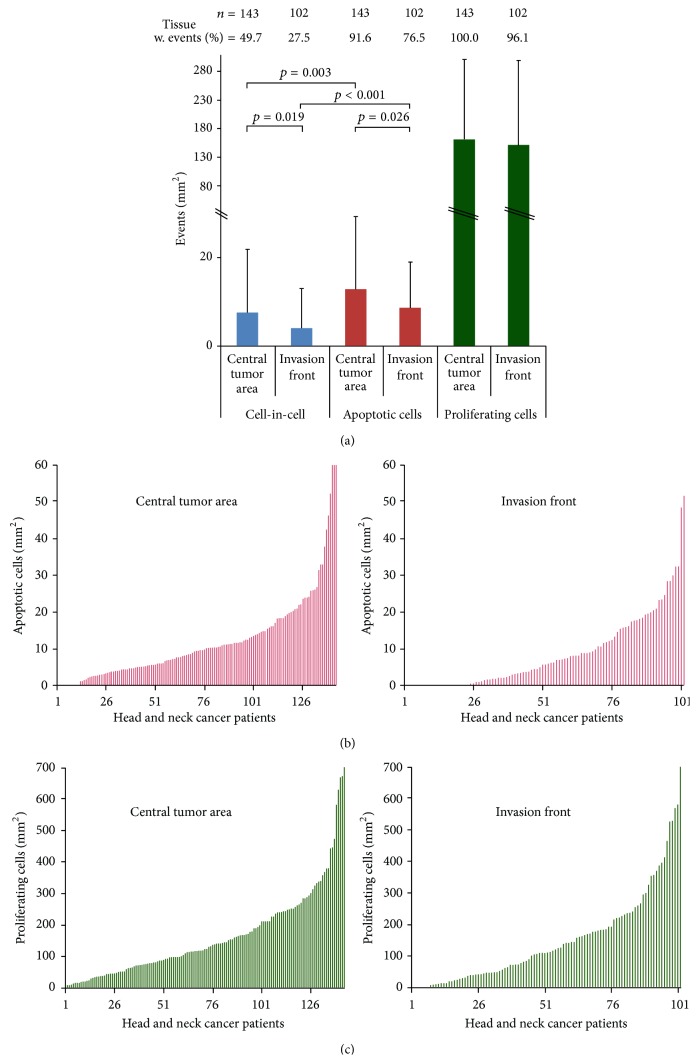
Apoptotic and proliferating cell rates. (a) CIC events, apoptotic cells, and proliferating cells in the central tumor area and invasive front of HNSCC. Frequency of (b) apoptotic and (c) proliferating cells in the central tumor area compared to the invasive front of individual HNSCC patients.

We then compared CIC frequency to apoptotic cell death and proliferation by quantifying apoptotic and proliferating cells in HNSCC samples stained for cleaved caspase-3 and Ki-67. In total, we studied 143 central tumor samples and 102 invasive front samples. Compared to invasive front samples, significantly more CIC structures and apoptotic cells were identified in samples from cancer centers. In the central tumor area, the portion of apoptotic cells was 1.6 times higher than that of CIC structures, and the number of apoptotic cells in the invasive front was two times higher (Figures 2(a) and 2(b)). The proportion of proliferating cells was much higher than that of CIC and apoptosis, with similar frequencies in the central tumor area and the invasive front (Figures 2(a) and 2(c)).

### 3.5. CIC Rate and Cell Death Events in Tumor Sections

**Figure 3:**
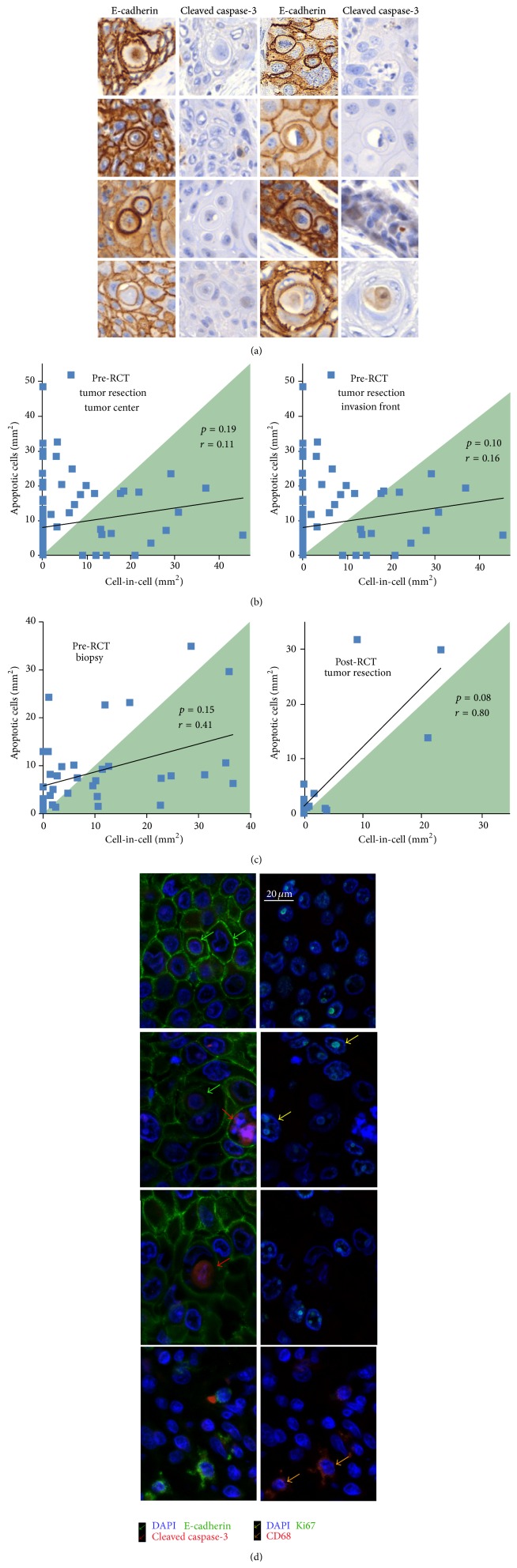
Cell-in-cell events compared to apoptotic events and prognostic impact of cell-in-cell events. (a) E-cadherin and cleaved caspase-3-labeled tumor sections containing CIC structures. (b) Frequency of CIC structures compared with the frequency of apoptotic cells in the central tumor area and invasive front of HNSCC. (c) CIC frequency compared to apoptotic cells in pretherapeutic biopsies and in posttherapeutic tumor resections of HNSCC. The shaded area marks tissues containing more CIC/mm^2^ than apoptotic cells/mm^2^. (d) Immunofluorescence staining for E-cadherin and cleaved caspase-3 in the left panel and Ki-67 and CD68 in the right panel. Nuclei were stained with DAPI.

We were particularly interested in whether the inner cells of CIC structures in tumor tissue were apoptotic. Thus, we stained the HNSCC tumor samples with cleaved caspase-3 to determine whether CIC structures in tumor tissue sections contained apoptotic cells. We counted 572 CIC events and found three cleaved caspase-3-positive cells; thus 99.5% of CIC structures were negative for cleaved caspase-3 (Figure 3(a)).

To determine whether there is a relationship between the frequency of CIC formation and apoptotic cells, we correlated both events in the pre- and posttherapeutic tissue samples from central tumor areas and invasion fronts. There was no correlation between these events, indicating that high numbers of apoptotic cells do not promote CIC formation (Figures 3(b) and 3(c)).

Additionally, we used multicolor immunofluorescence imaging of anal and HNSCC cancer in order to stain the tissue with DAPI, E-cadherin, cleaved caspase-3, Ki-67, and the phagocytic marker CD68. A recent report on homotypic cell cannibalism in pancreatic adenocarcinoma suggested ectopic expression of the scavenger receptor gene CD68 as a marker of cannibalistic cells in pancreatic adenocarcinoma [7]. Our histological study did not reveal expression of CD68 indicating that this molecule is not necessary for CIC formation in these tissues. We visualized rare CIC structures (green arrows) with inner apoptotic cells (red arrows), high numbers of not-engulfed Ki-67-positive proliferating cells (yellow arrow), and CD68-positive cells (orange arrows) that were presumably macrophages (Figure 3(d), additional file 1, Figure  A1).

### 3.6. CIC Rates as Prognostic Factor for Different Tumor Entities

**Figure 4:**
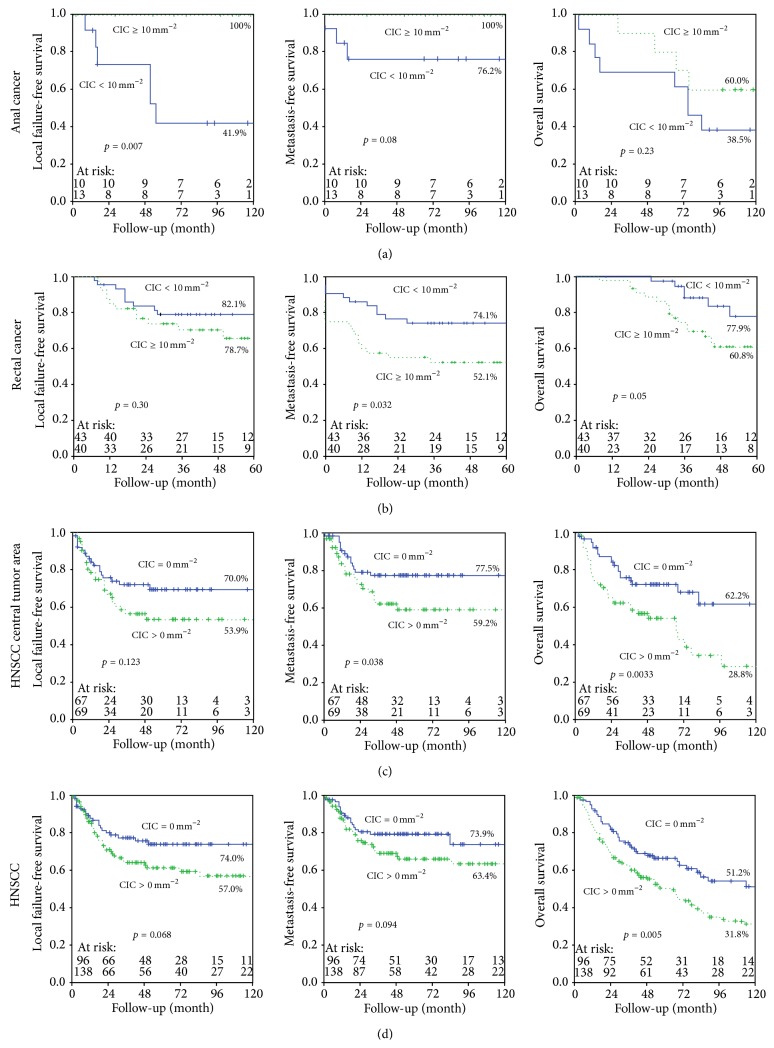
Kaplan-Meier analyses. (a) Kaplan-Meier curves depicting anal and (b) rectal cancer patients with fewer than 10 CIC structures per 10 mm^2^ (blue solid lines) and with equal or more than 10 CIC structures per 10 mm^2^ (green dotted lines). HNSCC patients with CIC structures (green dotted lines) and without CIC structures (blue solid lines) (c) in the central tumor area and (d) from all five HNSCC patient cohorts; from those patients the clinical data, patients' characteristics, and immunohistological data were completely available.

In total, 23 anal cancer patients, 83 rectal cancer patients, and 234 HNSCC patients were included in this analysis. Kaplan-Meier plot analysis was based on the elapsed time from the date of diagnosis, and this method revealed that more than 10 CICs/mm^2^ had a positive impact on local failure-free survival for anal cancer (*p* = 0.007). The correlation for metastasis-free, tumor-specific survival (additional file 1, Figure  A2) and overall survival was weaker. By contrast, either low numbers or the absence of CIC structures had a beneficial prognostic value in rectal cancer and HNSCC. In these cohorts, low CIC rates were associated with longer local failure-free and metastasis-free survival (*p* = 0.032, for rectal cancer). Low numbers or the absence of CIC structures was significantly correlated to an improved overall survival in HNSCC (*p* = 0.005) (Figure 4).

A multivariate analysis of the HNSCC adjuvant therapy subgroup with samples of the central tumor area was performed. Age, M-category, CIC/mm^2^, and proliferating cells/mm^2^ were included. Only CIC/mm^2^ and M-category were independent significant variables with impact on overall survival (Table 2). Neither apoptotic rates nor proliferating cells had an impact on overall survival. The five HNSCC cohorts were grouped and a multivariate analysis was performed; for those cases all clinical data, patient's characteristics, and immunohistological data were available. 234 patients were included and gender, M-category, grading, and CIC/mm^2^ were analyzed in a multivariate way. Again only CIC/mm^2^ and M-category were independent significant variables with impact on overall survival (additional file 2, Table  A2). In multivariate analysis of rectal cancer patients distance of tumor from anal verge, grading, and CIC/mm^2^ were included. Only grading was an independent significant variable with impact on overall survival (additional file 2, Table  A3).

## 4. Discussion

### 4.1. CIC Frequency in Tissue Correlates with Patient Outcomes

CIC events were observed in all of the cohorts, but the number of CIC-positive patients and the CIC rates vary considerably, depending on the cancer type, stage, previous therapeutic intervention, and localization of CIC events within the tumor. Compared to apoptotic cell rates, CIC rates are half to two-thirds as frequent. Though CICs are frequent events they have been nearly completely disregarded in the last 100 years [15]. We studied the possible correlation between CIC structures found in tumor tissues and patients' prognosis and confirmed that engulfed tumor cells in HNSCC and rectal and anal carcinoma tissues have a clear impact on the prognosis of these patients. In the anal cancer cohort, high numbers of CIC events led to an improved prognosis, whereas, in the HNSCC and rectal cancer cohorts, low numbers were associated with a good prognosis. Previously, a relationship between the occurrence of homotypic CIC structures and low risk for metastasis was observed in pancreatic adenocarcinoma patients [7]. In contrast, several studies have proposed a relationship between poor prognosis and the presence of homotypic cannibalism in breast carcinoma [6], bladder cancer [16], and medulloblastoma [5], as well as the presence of heterotypic cannibalism in gastric carcinoma [17]. Consistent with these heterogeneous studies, our results indicate that existence of CIC structures affects patient outcomes in different ways. The discrepancy observed in the correlation of CIC frequency and patient outcomes in different tumor entities might be explained by tissue and cell type-specific properties regarding tumor-specific metastatic potential and immune cell infiltration.

It was shown that homotypic CIC structures normally undergo cell death [7] regardless of whether alive cells or dead cells are internalized [4, 18]. Therefore, CIC seems to be a kind of cell death. However, the Nomenclature Committee on Cell Death (NCCD) demands the approval of CIC to be degraded within the cell as a result of homotypic interactions and that it should not be dependent on apoptotic cell [19]. Thus, we compared the frequency of apoptotic cells to CIC rates. We found CIC rates, which were half to two-thirds as frequent as apoptotic rates. However, only very rarely the engulfed cells were apoptotic and there was no correlation between the frequency of apoptotic and CIC rates. We hypothesize that the conditions of the NCCD are met. We would speculate that the engulfed cells were alive and matrix-deprived cells similar to metastatic cells and were phagocytized by cancer cells. Further, after engulfment cells lacked the chance to develop an apoptotic phenotype due to internalization and metabolic degradation.

Alternatively, it is possible that necrotic dying cells were phagocytized. Nevertheless, CIC frequency was an independent prognostic factor in multivariate analyses of the HNSCC cohorts, whereas apoptotic and proliferating rates did not reach a comparable impact. Maybe CICs have a similar high importance in prognosis as apoptosis and proliferation. Furthermore it could be speculated that CIC structure formation impacts tumor patient outcomes by elimination of detached cells that might form metastasis or suppression of the immune response by prohibition of emanation of immunogenic signals by nonapoptotic dying cells.

## 5. Conclusion

Our histological analyses showed that tumor cells containing incorporated, nonapoptotic tumor cells are commonly observed in many tumor tissue samples. Furthermore, we demonstrated that CIC structures correspond with clinical outcome depending on the cancer type and may have opposing effects on prognosis. CIC frequency is a valuable independent prognostic factor which should be considered in the same way as proliferation or apoptosis. Future work should also focus on functional studies in order to gain more insights into the mechanisms of CIC formation and to evaluate its potential value as a prognostic marker in other types of cancer.

## Supplementary Material

Figure A1: Two color fluorescence staining of a HNSCC tissue section. E-Cadherin is used to visualize cell-in-cell structures and cleaves caspase 3 to evaluate whether cell-in-cell cells are apoptotic.
Figure A2: Tumor specific survival in patients with anal cancer. It indicates that differences in anal cancers overall survival has other causes than tumor specific events.
Table A1: Patients 5 and 10 year survival time for HNSCC and anal cancer and 2.5 and 5 year for rectal cancer.
Table A2: Univariate and multivariate analysis from all five HNSCC patient cohorts.
Table A3: Univariate and multivariate analysis from the rectal cancer patients.

## Figures and Tables

**Table 1:** Clinical characteristics of the head and neck squamous cell carcinoma cohorts.

	All HNSCC		Early disease Adjuvant RCT			Advanced disease Definitive RT			Metastatic disease			Neoadjuvant RCT			Adjuvant RCT Center/invasion front		
*n*	321		62			52			29			35			143		
Gender (*n*)																	
Male	274	(85.4)	51	(82.3)	(15.9)	43	(82.7)	(13.4)	28	(96.6)	(8.7)	32	(91.4)	(10)	120	(83.9)	(37.4)
Female	47	(14.6)	11	(17.7)	(3.4)	9	(17.3)	(2.8)	1	(3.4)	(0.3)	3	(8.6)	(0.9)	23	(16.1)	(7.2)
Age (years)																	
Median (95% C.I.)	54.3	(53.9–55.7)	51.0	(49.4–53.3)		56.0	(53.2–58)		53.0	(49.3–55)		52.5	(51.3–54.5)		58.0	(56.2–59.2)	
Local failure time (months)																	
Median (95% C.I.)	50.0	(58.3–69.4)	67.0	(72.1–111)		42.0	(44.9–72.4)		51.0	(48.8–83.9)		87.0	(74.1–96.6)		32.0	(34.5–46.1)	
Time to metastatic disease (months)																	
Median (95% C.I.)	53.0	(61.7–73)	81.5	(82–121.7)		42.0	(44.7–72.4)		61.0	(51.3–85.7)		90.0	(77.2–99.6)		37.5	(37–48.3)	
Overall survival time (months)																	
Median (95% C.I.)	54.0	(63.5–74.6)	81.5	(82–121.7)		44.0	(47.6–74.2)		61.0	(53.8–89.3)		90.0	(77.5–99.8)		41.0	(39.9–51)	
T stage (*n*)																	
T1	59	(18.4)	13	(21)	(4)	0	0	(0)	7	(24.1)	(2.2)	3	(8.6)	(0.9)	36	(25.2)	(11.2)
T2	105	(32.7)	30	(48.4)	(9.3)	2	(3.8)	(0.6)	8	(27.6)	(2.5)	9	(25.7)	(2.8)	56	(39.2)	(17.4)
T3	78	(24.3)	14	(22.6)	(4.4)	25	(48.1)	(7.8)	6	(20.7)	(1.9)	6	(17.1)	(1.9)	27	(18.9)	(8.4)
T4	79	(24.6)	5	(8.1)	(1.6)	25	(48.1)	(7.8)	8	(27.6)	(2.5)	17	(48.6)	(5.3)	24	(16.8)	(7.5)
N stage (*n*)																	
N0	68	(21.2)	23	(37.1)	(7.2)	5	(9.6)	(1.6)	0	(0)	(0)	7	(20.0)	(2.2)	38	(26.6)	(11.8)
N1	55	(17.1)	17	(27.4)	(5.3)	4	(7.7)	(1.2)	4	(13.8)	(1.2)	2	(5.7)	(0.6)	29	(20.3)	(9)
N2	186	(57.9)	22	(35.5)	(6.9)	41	(78.8)	(12.8)	23	(79.3)	(7.2)	25	(71.4)	(7.8)	69	(48.3)	(21.5)
N3	12	(3.7)	0	(0)	(0)	2	(3.8)	(0.6)	2	(6.9)	(0.6)	1	(2.9)	(0.3)	7	(4.9)	(2.2)
M stage (*n*)																	
M0	237	(73.8)	62	(100.0)	(19.3)	39	(75.0)	(12.1)	6	(20.7)	(1.9)	32	(91.4)	(10)	98	(68.5)	(30.5)
M1	84	(26.2)	0	(0.0)	(0)	13	(25.0)	(4)	23	(79.3)	(7.2)	3	(8.6)	(0.9)	45	(31.5)	(14)
Grading (*n*)																	
G1/2	176	(54.8)	37	(59.7)	(11.5)	37	(71.2)	(11.5)	15	(51.7)	(4.7)	27	(77.1)	(8.4)	60	(42)	(18.7)
G3/4	145	(45.2)	25	(40.3)	(7.8)	15	(28.8)	(4.7)	14	(48.3)	(4.4)	8	(22.9)	(2.5)	83	(58)	(25.9)
UICC97 (*n*)																	
1	15	(4.7)	6	(9.7)	(1.9)	0	(0)	(0)	0	(0)	(0)	1	(2.9)	(0.3)	8	(5.6)	(2.5)
2	30	(9.3)	13	(21)	(4)	0	(0)	(0)	1	(3.4)	(0.3)	2	(5.7)	(0.6)	14	(9.8)	(4.4)
3	61	(19)	17	(27.4)	(5.3)	7	(13.5)	(2.2)	2	(6.9)	(0.6)	2	(5.7)	(0.6)	33	(23.1)	(10.3)
4	215	(67)	26	(41.9)	(8.1)	45	(86.5)	(14)	26	(89.7)	(8.1)	30	(85.7)	(9.3)	88	(61.5)	(27.4)

Values behind the first bracket are relative values within the subcohort and values behind the second bracket are relative values of the total cohort. Confidence interval: C.I.

**Table 2:** Clinical characteristics of the rectal and anal cancer cohorts.

	Rectal cancer, clinical	Rectal cancer, pathological	Anal cancer
All	83	83	23
Male	59 (71.1)		9 (39.1)
Female	24 (28.9)		14 (60.9)
Age (years)			
Median (95% C.I.)	64.0 (59.7–65.2)		59.0 (48.7–64.2)
Local failure time (months)			
Median (95% C.I.)	39.0 (35.7–44.1)		71.0 (53.3–98.8)
Time to metastatic disease (months)			
Median (95% C.I.)	38.0 (29.9–40.5)		82.0 (62–113.5)
Overall survival time (months)			
Median (95% C.I.)	44.0 (41.9–48.6)		86.5 (65.9–115.6)
T stage (*n*)			
T0	—	11 (13.3)	—
T1	0 (0)	2 (2.4)	1 (4.3)
T2	7 (8.4)	25 (30.1)	13 (56.5)
T3	64 (77.1)	32 (38.6)	7 (30.4)
T4	12 (14.5)	13 (15.7)	2 (8.7)
N stage (*n*)			
N0	18 (21.7)	53 (63.9)	10 (43.5)
N1	52 (62.7)	30 (36.1)	5 (21.7)
N2	13 (15.7)	0 (0.0)	8 (34.8)
M stage (*n*)			
M0	53 (63.9)	—	20 (87)
M1	30 (36.1)	—	3 (13)
Grading (*n*)			
G1/2	73 (88.0)		16 (69.6)
G3/4	10 (12.0)		7 (30.4)
UICC97 (*n*)			
1	22 (26.5)		3 (13)
2	28 (33.7)		7 (30.4)
3	21 (25.3)		13 (56.5)
4	12 (14.5)		0 (0)

**Table 3:** Univariate and multivariate overall survival analyses according to Cox's proportional hazards model and HNSCC adjuvant radiochemotherapy central tumor area collectively.

HNSCC central tumor area; *n* = 143	Univariate analysis			Multivariate analysis		
Variable	Hazard ratio	95% C.I.	*p*	Hazard ratio	95% C.I.	*p*
Age, years (younger than 58 years [*n* = 72] versus older than 58 years [*n* = 71])	1.853	1.007–3.41	**0.048 **	1.559	0.901–2.697	0.113
Gender (male [*n* = 120] versus female [*n* = 23])	1.275	0.566–2.871	0.558	—	—	—
T category (T1/T2 [*n* = 92] versus T3/T4 [*n* = 51])	0.748	0.36–1.554	0.436	—	—	—
N category (N0 [*n* = 38] versus N+ [*n* = 105])	1.080	0.333–3.5	0.899	—	—	—
M-category (M0 [*n* = 98] versus M+ [*n* = 45])	8.094	1.59–41.214	**0.012 **	4.660	1.377–15.767	**0.013 **
Stage (UICC I [*n* = 22] versus UICC II and higher [*n* = 121])	0.971	0.309–3.053	0.960	—	—	—
Grade (1 + 2 [*n* = 60] versus 3 + 4 [*n* = 83])	0.987	0.526–1.851	0.967	—	—	—
CIC (0/mm² [*n* = 74] versus >0/mm² [*n* = 69])	2.555	1.334–4.894	**0.005 **	2.139	1.215–3.764	**0.008 **
Apoptotic cells (<9.5/mm² [*n* = 71] versus ≥9.5/mm² [*n* = 72])	1.037	0.329–3.266	0.950	—	—	—
Proliferating cells (<122/mm² [*n* = 71] versus ≥122/mm² [*n* = 72])	0.689	0.358–1.325	0.264	0.686	0.39–1.208	0.192
