# Supplementary material for: Prognostic Value of Homotypic Cell Internalization by Nonprofessional Phagocytic Cancer Cells
Source: Biomed Res Int. 2015 Oct 4;2015:359392. doi: 10.1155/2015/359392 (PMC4609350; doi:10.1155/2015/359392)
Supplement: Supplementary file 1 — Figure A1: Two color fluorescence staining of a HNSCC tissue section. E-Cadherin is used to visualize cell-in-cell structures and cleaves caspase 3 to evaluate whether cell-in-cell cells are apoptotic. Figure A2: Tumor specific survival in patients with anal cancer. It indicates that differences in anal cancers overall survival has other causes than tumor specific events. Table A1: Patients 5 and 10 year survival time for HNSCC and anal cancer and 2.5 and 5 year for rectal cancer. Table A2: Univariate and multivariate analysis from all five HNSCC patient cohorts. Table A3: Univariate and multivariate analysis from the rectal cancer patients. [file 359392.f1.zip › Additional final 1.docx]

**Additional file 1.
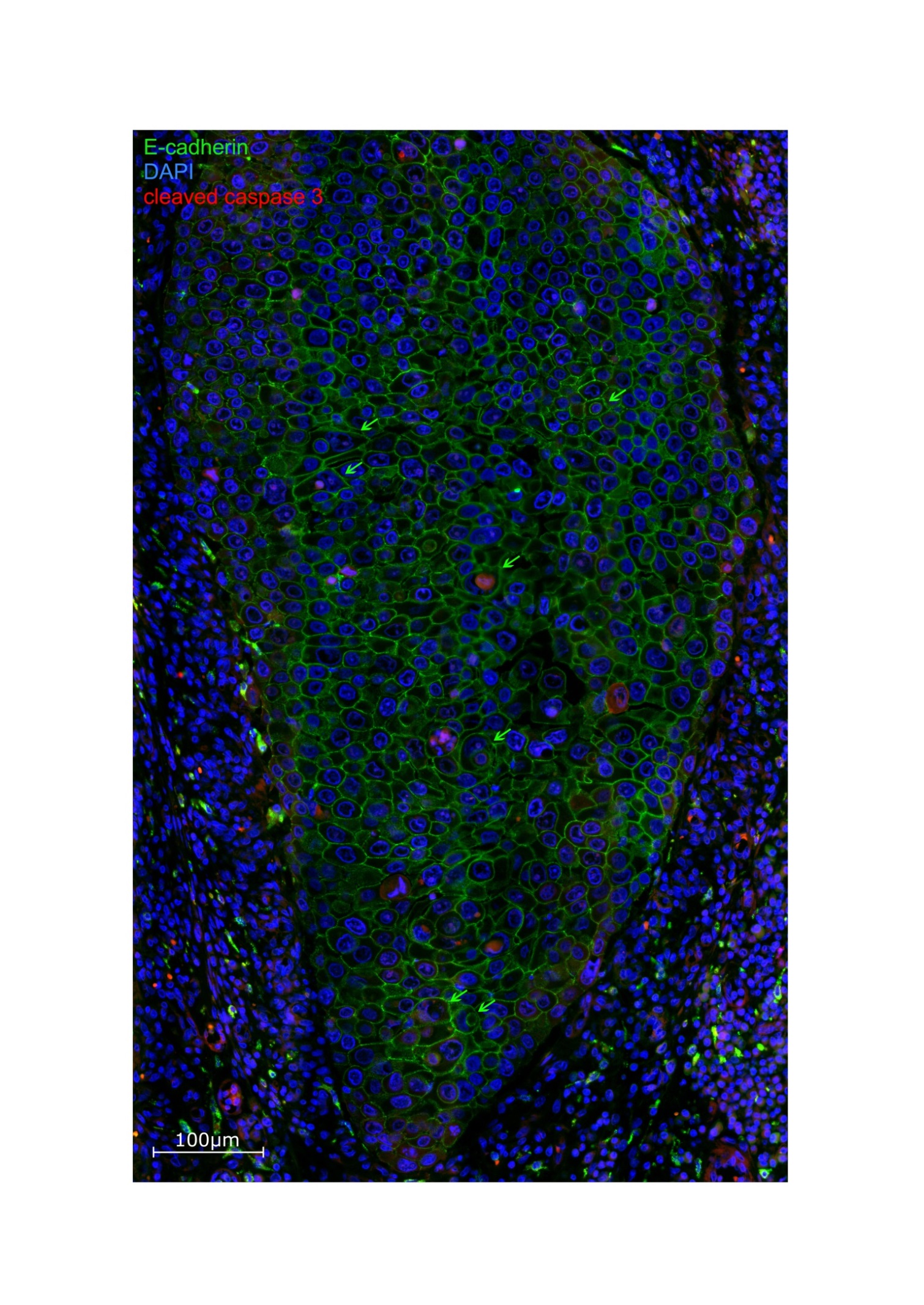
**

**Figure A1: CIC structures occur frequently in tumor sections.**

Immunofluorescence staining for E-cadherin (green) and cleaved caspase-3 (red) in a representative HNSCC tissue section. Nuclei were stained blue with DAPI. The green arrows indicate typical CIC structures.


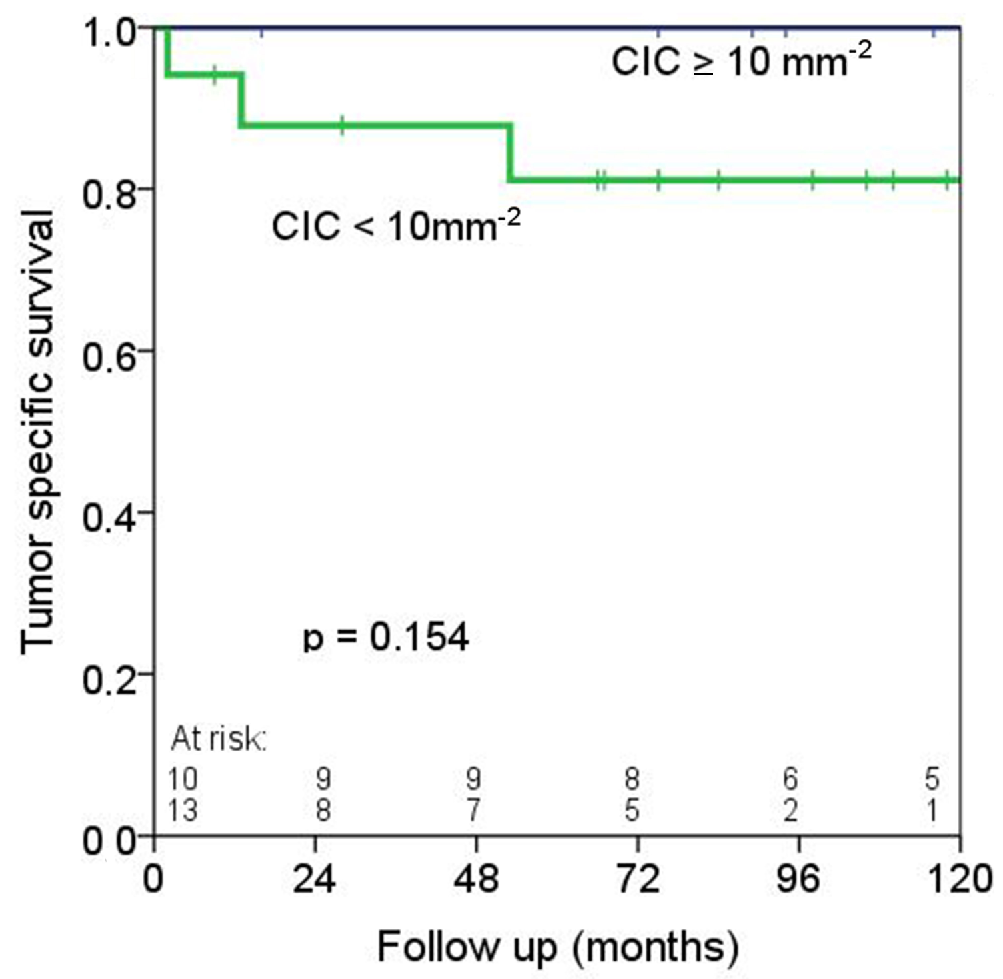


**Figure A2: Tumor specific survival in patients with anal cancer**

Kaplan-Meier curves of tumor specific survival depicting anal cancer patients with fewer than 10 CIC structures per 10 mm^2^ (green lines) and with equal or more than 10 CIC structures per 10 mm^2^ (blue lines).
