# Supplementary material for: Prognostic Value of Homotypic Cell Internalization by Nonprofessional Phagocytic Cancer Cells
Source: Biomed Res Int. 2015 Oct 4;2015:359392. doi: 10.1155/2015/359392 (PMC4609350; doi:10.1155/2015/359392)
Supplement: Supplementary file 1 — Figure A1: Two color fluorescence staining of a HNSCC tissue section. E-Cadherin is used to visualize cell-in-cell structures and cleaves caspase 3 to evaluate whether cell-in-cell cells are apoptotic. Figure A2: Tumor specific survival in patients with anal cancer. It indicates that differences in anal cancers overall survival has other causes than tumor specific events. Table A1: Patients 5 and 10 year survival time for HNSCC and anal cancer and 2.5 and 5 year for rectal cancer. Table A2: Univariate and multivariate analysis from all five HNSCC patient cohorts. Table A3: Univariate and multivariate analysis from the rectal cancer patients. [file 359392.f1.zip › Additional final 2.docx]

**Table A1: Patients’ survival**

|  | HNSCC all | | Anal cancer | | Rectal cancer | |
| --- | --- | --- | --- | --- | --- | --- |
|  | 5 years | 10 years | 5 years | 10 years | 2.5 years | 5 years |
| Local failure-free survival | 71.6% | 66.6% | 70.9% | 70.9% | 76.3% | 72.2% |
| Overall survival | 60.3% | 39.5% | 75.0% | 50.0% | 90.2% | 68.5% |
| Metastasis-free survival | 81.1% | 78.8% | 87.3% | 87.3% | 64.8% | 63.4% |
| No evidence of disease | 69.3% | 63.9% | 68.0% | 68.0% | 63.6% | 60.0% |

Patients 5 and 10 year survival time for HNSCC and anal cancer and 2.5 and 5 year for rectal cancer.

**Table A2: Univariate and multivariate overall-survival analyses according to Cox's proportional hazards model, HNSCC.**

| HNSCC, n = 234 | Univariate analysis | | | Multivariate analysis | | |
| --- | --- | --- | --- | --- | --- | --- |
| Variable | Hazard ratio | 95% C.I. | p | Hazard ratio | 95% C.I. | p |
| Age, years (younger 54 years [n = 117] v. older 54 years [n = 117]) | 1.093 | 0.76 - 1.572 | 0.632 | --- | --- | --- |
| gender (male [n = 199] v. female [n = 35]) | 0.659 | 0.406 - 1.072 | 0.093 | 0.698 | 0.44 - 1.107 | 0.127 |
| T category (T1/T2 [n = 150] v. T3/T4 [n = 84]) | 1.079 | 0.73 - 1.597 | 0.702 | --- | --- | --- |
| N category ( N0 [n = 61] v. N+ [n = 173]) | 0.932 | 0.462 - 1.879 | 0.844 | --- | --- | --- |
| M category (M0 [n = 166] v. M+ [n = 68]) | 3.480 | 1.961 - 6.175 | **<0.001** | 3.434 | 2.036 - 5.793 | **< 0.001** |
| Stage (UICC I [n = 42] v. UICC II and higher [n = 192]) | 1.008 | 0.489 - 2.078 | 0.982 | --- | --- | --- |
| Grade (1+2 [n = 112] v. 3+4 [n = 122] ) | 1.124 | 0.781 - 1.617 | 0.529 | 1.101 | 0.775 - 1.566 | 0.591 |
| CIC (0 [n = 96] v. > 0 /mm² [n = 138]) | 1.642 | 1.127 - 2.392 | **0.010** | 1.562 | 1.093 - 2.232 | **0.014** |

Univariate and multivariate analysis from all five HNSCC patient cohorts from those patients the clinical data, patients’ characteristics and immunohistological data were available. Univariate and multivariate overall-survival analyses according to Cox's proportional hazards model were performed. The proportional hazards assumption was tested by visual inspection of log minus log curves, and was found to be satisfied for all multivariate covariates.

**Table A3:** **Univariate and multivariate overall-survival analyses according to Cox's proportional hazards model, rectal cancer.**

| Rectal cancer, n = 83 | Univariate analysis | | | Multivariate analysis | | |
| --- | --- | --- | --- | --- | --- | --- |
| Variable | Hazard ratio | 95% C.I. | p | Hazard ratio | 95% C.I. | p |
| Age, years (younger 64 years [n = 42] v. older 64 years [n = 41]) | 0.709 | 0.271 - 1.856 | 0.484 | --- | --- | --- |
| Gender (male [n = 59] v. female [n = 24]) | 1.640 | 0.629 - 4.278 | 0.312 | --- | --- | --- |
| Distance of tumor from anal verge (≤ 7.5cm [n = 40] v. > 7.5cm [n = 43]) | 0.389 | 0.15 - 1.00 | **0.050** | 0.559 | 0.238 - 1.314 | 0.182 |
| cT category (≤ T3 [n = 71] v. T4 [n = 12]) | 0.547 | 0.104 - 2.867 | 0.475 | --- | --- | --- |
| cN category (N0 [n = 18] v. N+ [n = 65]) | 2.320 | 0.618 - 8.71 | 0.213 | --- | --- | --- |
| pT category (≤ T3 [n = 70] v. T4 [n = 13]) | 1.595 | 0.568 - 4.479 | 0.376 | --- | --- | --- |
| pN category (N0 [n = 53] v. N+ [n = 30]) | 0.520 | 0.105 - 2.572 | 0.422 | --- | --- | --- |
| L category (L0 [n = 67] v. L+ [n = 16]) | 1.119 | 0.267 - 4.688 | 0.878 | --- | --- | --- |
| V category (V0 [n = 76] v. V+ [n = 7]) | 0.460 | 0.065 - 3.284 | 0.439 | --- | --- | --- |
| Stage (UICC I [n = 50] v. UICC II and higher [n = 33]) | 1.027 | 0.218 - 4.826 | 0.973 | --- | --- | --- |
| Grade (1+2 [n = 73] v. 3+4 [n = 10]) | 4.595 | 0.999 - 21.298 | **0.050** | 4.143 | 1.685 - 10.188 | **0.002** |
| CIC (< 10 [n = 39] v. ≥ 10 /mm² [n = 44]) | 2.024 | 0.745 - 5.503 | 0.167 | 2.038 | 0.79 - 5.257 | 0.141 |

Human specimen of rectal cancer patients and univariate and multivariate overall-survival analyses according to Cox's proportional hazards model. The proportional hazards assumption was tested by visual inspection of log minus log curves, and was found to be satisfied for all multivariate covariates.
